# Deficiency of microRNA-628-5p promotes the progression of gastric cancer by upregulating PIN1

**DOI:** 10.1038/s41419-020-02766-6

**Published:** 2020-07-23

**Authors:** Yang Chen, Yaran Wu, Shuhui Yu, Hongying Yang, Xiya Wang, Yali Zhang, Shunqin Zhu, Mengmeng Jie, Cheng Liu, Xinzhe Li, You Zhou, Shiming Yang, Yingbin Yang

**Affiliations:** 1 School of Life Science, Southwest University. No. 1, Tiansheng RD, Beibei District, Chongqing, 400715 China; 2 Department of Gastroenterology, Xinqiao Hospital, Third Military Medical University. No. 183, Xinqiao Main ST, Shapingba District, Chongqing, 400037 China; 3 Department of Hematology, Southwest Hospital, Third Military Medical University. No. 30, Gaotanyan Main ST, Shapingba District, Chongqing, 400038 China; 4 College of Biotechnology, Southwest University. No. 1, Tiansheng RD, Beibei District, Chongqing, 400715 China

**Keywords:** Oncogenes, Gastrointestinal cancer

## Abstract

Gastric cancer is one of the most common cancer and is the second leading cause of cancer-related mortality in the world. PIN1, belonging to peptidyl-prolyl cis-trans isomerase family, uniquely catalyzes the structural transformation of phosphorylated Ser/Thr-Pro motif. It’s high expressed in most cancers and promotes their progression. However, the mechanism of PIN1 high expression and its function in gastric cancer progression are still unclear. In this research, we revealed that PIN1 not only promotes the proliferation and colony formation of gastric cancer, but also increases its migration and invasion. The PIN1 expression in metastasis lesion is usually higher than the corresponding primary site. Inhibiting PIN1 by shRNA suppresses the progression of gastric cancer significantly. Besides, we demonstrated that miR-628-5p is a novel PIN1-targeted microRNA, and the expression of miR-628-5p is negatively correlated with PIN1 in gastric cancer. Exogenous expression of miR-628-5p inhibits the progression of gastric cancer that revered by restoring PIN1 expression. However, miR-628-5p is downregulated in majority of gastric cancer tissue especially in metastasis lesion. The lower miR-628-5p level indicates poorer prognosis. In summary, our study demonstrated that deficient miR-628-5p expression facilitates the expression of PIN1, and consequently promotes the progression of gastric cancer.

## Introduction

The incidence and mortality of gastric cancer are one of the highest in the world. In recent years, with the improvement of diagnosis and treatment technology, the gastric cancer incidence and mortality rate are significantly decreased. However, patients that diagnosed in advanced stages still have a high risk of poor prognosis. Therefore, it’s of great significance to illuminate the mechanism of gastric cancer progression.

Peptidyl-prolyl cis-trans isomerase NIMA-interacting 1 (PIN1) is a member of the peptidyl-prolyl cis-trans isomerases (PPIases) superfamily. It uniquely catalyzes conformational transformation of the phosphorylated serine/threonine-proline (pSer/Thr-Pro) motif^1–3^. Ser/Thr-Pro motif widely exist in various proteins that makes PIN1 vital in many cellular processes by regulating multiple signaling pathways^4^. Our previous study have demonstrated that the mutant of Ser246-Pro247 motif in β-catenin leads to its subcellular localization variation^5^. Aberrant PIN1 expression or activation leads to both degenerative disorders and cancers^6^. In most cancers, PIN1 is significantly high expressed and correlates with poor prognosis^7^. It facilitates multiple cancer-driving signaling pathways and contributes to all the major ten biological capabilities of cancers^8^.

Recently, researches indicated that PIN1 also high expressed in gastric cancer that promotes its proliferation^9,10^ and chemoresistance^11^. In this work, we demonstrated that PIN1 also facilitates the migration and invasion of gastric cancer. It’s high expressed in most of cancer tissues and even higher in metastasis lesions. However, the mechanism of PIN1 high expression in gastric cancer is still unclear. MicroRNAs (miRs) regulated the expression of genes at both transcriptional and posttranscriptional levels^12^. Researches have revealed that miR-200b-3p^13^, miR-200c-3p^14^, miR-296-5p^15^, miR-874-3p^16^, miR-370-3p^17^, and miR-140-5p^18^ inhibit PIN1 in different cancers but gastric cancer.

To reveal PIN1-targeted miR in gastric cancer, we predicted the potential miRs at five databases, including miRTarBase, TargetScan, miRNApath, starBase, and miRanda. Thirty six candidates appeared in three or more databases, including the six reported miRs. To identify novel PIN1-targeted miR, we then detected whether the thirty unreported miRs suppress PIN1 expression in gastric cancer. Result shows that miR-122-5p, miR-331-3p, miR-346, miR-628-5p, and miR-760 inhibit PIN1 mRNA expression in at least three gastric cell lines, meanwhile, miR-628-5p suppresses its protein expression most significantly. Our following study illuminated that miR-628-5p binds to the 3′UTR of PIN1 mRNA directly. Researches indicated that miR-628-5p inhibits prostate cancer^19^, glioma^20^, and epithelial ovarian cancer^21^ by targeting different genes, but it’s usually downregulated in these cancers. In this work, we firstly demonstrated that miR-628-5p inhibits gastric cancer by targeting PIN1. However, the miR-628-5p expression is also deficient in majority of gastric cancer tissues especially in metastasis tissues and lower miR-628-5p indicates poorer prognosis. According to these results, we confirmed that deficient miR-628-5p expression promotes gastric cancer by upregulating PIN1.

## Materials and methods

### Human tissue specimens

All gastric cancer tissues and paired adjacent tissues for quantitative real-time polymerase chain reaction (qRT-PCR) and western blot validation were collected from the Department of General Surgery of the Southwest and Xinqiao Hospital, Third Military Medical University. All tissues were immediately preserved in liquid nitrogen. Written informed consent was obtained from all patients and the study was approved by the Ethics Committee of the Second Affiliated Hospital of Third Military Medical University (ChiCTR1900026337). The human tissue microarrays of human gastric cancer tissues and corresponding adjacent noncancerous tissues, primary and metastasis tissues, were purchased from Shanghai Outdo Biotech. Co. Ltd. (Shanghai, China).

### Cell culture and reagents

AGS, HGC-27, and GES-1 cell lines were obtained from American Type Culture Collection (Manassas, Virginia, USA), MKN-45, MKN-74, and MKN-28 were from JCRB Cell Bank (National Institute of Hygienic Sciences,Tokyo), BGC-823 and SGC-7901 cell lines were purchased from Cell Bank of the Shanghai Institute for Biological Sciences (Chinese Academy of Sciences, Shanghai, China), and MGC-803 was from National Infrastructure of Cell Line Resource (Beijing, China). All cell lines were genotyped for identity by Shanghai Biowing Applied Biotechnology Co., Ltd. and tested routinely for Mycoplasma contamination. HGC-27, MKN-28, MKN-74, SGC-7901, MGC-803, and BGC-823 cells were maintained in Dulbecco’s Modified Eagle Medium (Hyclone) while GES, AGS, and MKN-45 were cultured in RPMI 1640 medium (Hyclone). All medium were supplemented with 10% fetal bovine serum (Hyclone) and penicillin–streptomycin (penicillin 100 U/ml and streptomycin 0.1 mg/ml, Beyotime). All cell lines were cultured in a 37 °C incubator supplemented with humidified and 5% CO_2_ atmosphere.

### Plasmids, miR, inhibitor, and transfection

The cDNA from gastric cancer cell was used as template to amplify PIN1 CDS (the primer sequence were shown in Supplementary Table 1). The PIN1 CDS was subcloned to the pCMV5 vector with Kpn I and Hind III endonucleases. The PIN1 shRNA plasmid (target sequence: CGCAAAGGTGAACACTCATGC) was purchased from GeneCopoeia. The wild-type PIN1 3′UTR reporter plasmid (psiCheck2-PIN1-3′UTR-WT) was purchased from Sangon Biotech and the sequence were shown in Supplementary Table 2. Based on this plasmid, the mutant reporter plasmids were constructed by high-fidelity DNA polymerase (NEB) with different mutant primers (Supplementary Table 1). The miR mimics and inhibitors were synthesized from Sangon Biotech. The plasmids, mimics, or inhibitors was transfected by Lipofectamine 3000 reagent (Thermo Fisher) and detect at 48 h post transfection.

### Reverse transcription and qRT-PCR

Total RNA was extracted by Trizol reagent (TaKaRa). A total of 1 μg total RNA was reverse transcribed to cDNA by PrimeScript RT reagent Kit (TakaRa). Oligo dT and random primer mix were used for mRNA reverse transcription, while gene-specific reverse transcription primers were used for miR and U6 reverse transcription. The cDNAs were quantified by qPCR with TB Green Premix Ex Taq mix (TakaRa). The primer sequence were shown in Supplementary Table 1.

### Protein extraction and western blotting

Cells were collected and lysed by RIPA lysis buffer (Beyotime), put on ice for 20 min and harvested the supernatant after centrifuged at 12,000 × *g*, 4 °C for 20 min. The concentration of protein samples were detected by BCA Protein Assay Kit (Beyotime). Then, the protein samples were standardized, subjected to SDS–PAGE, transferred to PVDF membrane (Millipore), immunoblotted, and exposed by ECL (Thermo). Primary antibodies of anti-PIN1 (10495-1-AP) and anti-LaminB1 (12987-1-AP) were purchased from Proteintech. Anti-cyclin D1 (55506 S), anti-MMP2 (40994 S), anti-MMP9 (13667 S), anti-β-catenin (8480 S), anti-E-cadherin (14472 S), anti-N-cadherin (13116 S), and anti-XPO5 (12565 S) were from CST. The β-actin expression was applied as control for normalization (CST, 3700). The HRP-conjugated goat anti-rabbit IgG antibody (ZB-2301) and HRP-conjugated goat anti-mouse IgG antibody (ZB-2305) were from ZSGB-Bio.

### Proliferation assay

Treated AGS, HGC-27, SGC-7901, or MGC-803 were collected, resuspended, counted, and diluted to 1.5 × 10^4^ cells per milliliter (1.5 × 10^4^ cells/ml) with complete medium. A total of 200 μl of each cells were plated into 96-well plates (at least three replicas). The cell viability were detected by CCK-8 assay (Beyotime) at indicated time points.

### Flow cytometry

Treated cells were trypsinized and fixed in 70% ethanol at 4 °C for 24 h, then the cells were incubated with propidium iodide (40 μg/ml; Sangon Biotech) and RNase A (100 μg/ml; TakaRa) in PBS at 37 °C for 1 h. Cells were centrifuged for 5 min, resuspended in the PBS, and analyzed using a flow cytometer (BD).

### Colony formation

Treated cells were collected, resuspended, and counted. A total of 500 cells suspended in 3 ml complete medium were plated in a well of six‐well plates, then incubated in the cell incubator for 10–14 days according to the colony size. The cells were fixed with absolute methanol for 10 min and stained with crystal violet (Beyotime) for 20 min. The colonies were photographed and counted. All the experiments were performed at least triplicate wells.

### Migration and invasion assay

Transwell assay were performed in 24-well cell culture inserts (8.0 μm pore size, Millipore) and the 24-well plates was pre-added 0.5 ml complete medium. Treated cells were collected, resuspended with serum-free medium, counted, and diluted to a certain concentration. For migration, 4–5 × 10^4^ cells diluted in 200 μl serum-free medium were added to each transwell insert. For invasion, the inserts were pre-added 80 μl of diluted matrigel (BD, cat. no.: 356234, matrigel: medium = 1:8). Then, 1.0–1.5 × 10^5^ cells diluted in 200 μl serum-free medium were added to each insert.

The penetrated cells were detected at 24 h postinoculation. Remove the cells in the upper part of the insert with cotton swab, fix the cells with 4% PFA (Boster), and stain with crystal violet, filters were photographed at five random fields and the total penetrated cells of each field were counted. The means ± standard deviation (s.d.) were showed in the final result. All the experiments were repeated at least three times.

### RNA-binding protein immunoprecipitation

RNA-binding Protein Immunoprecipitation (RIP) Kit (Millipore, cat. no.:17-700) was applied for this assay. MGC-803 or AGS were transfected with 200 nM miR mimics or inhibitor respectively. A total of 2 × 10^7^ cells were collected and lysed. Remove 10 μl of supernatant into a new tube as input. A total of 5 μg of AGO2 antibody was used for each immunoprecipitation. Incubate with ratting overnight at 4 °C. Purification the precipitated RNA and detected the abundance of PIN1 mRNA by qRT-PCR.

### Luciferase reporter assay

A total of 40–50% integrated SGC-7901 were prepared for transfection. A total of 200 nM miR mimics or inhibitors was cotransfected with psiCheck2-PIN1-3′UTR-WT or different mutant plamids. The relative luciferase activities were detected at 48 h post transfection by Dual-luciferase reporter assay (Promega). All the experiments were repeated at least three replicas.

### In situ hybridization

The tissue chip was purchased from Shanghai Outdo biotech and enhanced sensitive in situ hybridization (ISH) detection kit (Boster) was applied. Hybridization was carried out at 40 °C overnight with 4 μM digoxin-labeled probes (Sangon Biotech) in a humidified condition. The stained tissues were photographed by microscope and quantified by image-Pro Plus 6.0 software.

### Immunohistochemistry

Immunohistochemistry (IHC) kit (Boster) was applied. The tissue chip was purchased from Shanghai Outdo biotech and antigens repaired by boiling water bath. The PIN1 antibody (Proteintech) was incubated at 4 °C overnight. The stained tissues were photographed by microscope and quantified by image-Pro Plus 6.0 software.

### Statistical analyses

The expression of PIN1 and miR-628-5p between 24 pairs of gastric cancer and adjacent normal cancer tissues were analyzed by ggplot2. The correlation coefficients were calculated by Pearson’s rank correlation test. The Kaplan–Meier method was used for survival analysis. For comparisons between two groups, Student’s *t*-test was applied if no significantly different variances existed. When more than two groups were compared, one-way analysis of variance (ANOVA) was performed followed by Tukey’s test. To calculate the *p* value between groups in Fig. 6, Supplementary Fig. 4, two-way ANOVA analysis was performed with Prism 8. *p < 0.05, **p < 0.01, ***p < 0.001, ns = no significance. p < 0.05 was considered statistically significant.

## Result

### PIN1 is upregulated in gastric cancer and indicates poorer prognosis

**Fig. 1:**
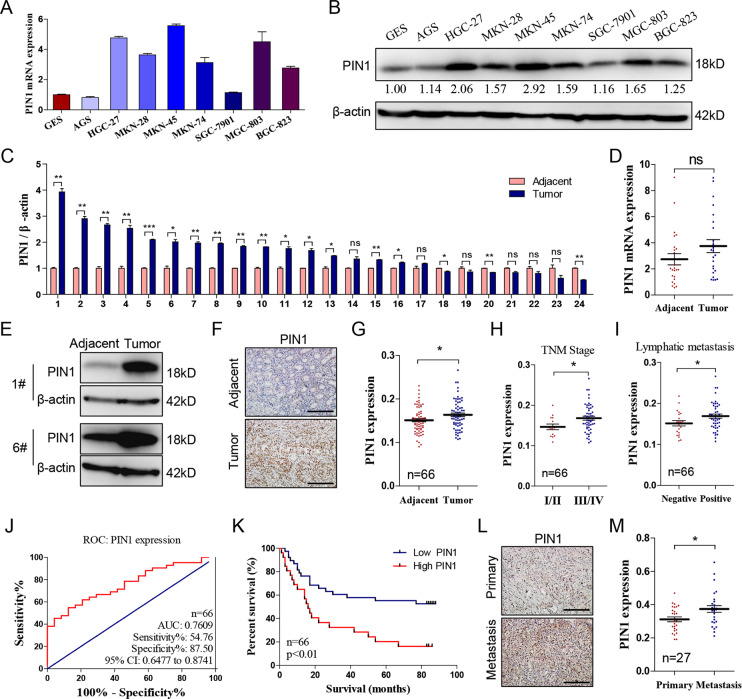
**PIN1 is upregulated in gastric cancer and indicates poorer prognosis.** **a**, **b** The mRNA and protein level of PIN1 in different gastric cancer cell lines and human GES. The relative protein densitometry was analyzed. **c**, **d** The PIN1 mRNA expression in the cancer and corresponding adjacent tissue (*n* = 24, *p < 0.05, **p < 0.01, ***p < 0.001, ns, no significance). **e** Representative protein expression of PIN1 from tissues above. **f** Representative immunohistochemical staining of PIN1 in the cancer and adjacent tissue. **g** The PIN1 expression in cancer and adjacent tissue (*n* = 66, *p < 0.05). **h**, **i** The correlation of PIN1 expression with TNM stage/lymphatic metastasis in gastric cancer (*n* = 66, *p < 0.05). **j** The ROC curves based on PIN1 expression to predict patients’ survival time. **k** The PIN1 expression based overall survival of gastric cancer patient by Kaplan–Meier analyses (*n* = 66, *p* < 0.01). **l** Representative immunohistochemical staining of PIN1 in the primary and metastasis cancer tissues. **m** The PIN1 expression in primary and metastasis cancer tissues (*n* = 27, *p < 0.05). Scale bars: 200 μm.

Researches have indicated that PIN1 is upregulated in multiple cancers including gastric cancer. In our research, we detected the mRNA and protein level of PIN1 in eight gastric cancer cell lines and human gastric epithelium cell line (GES). Meanwhile, we also determined its mRNA expression in 24 pairs of gastric cancer and corresponding adjacent tissues. Result revealed that PIN1 is upregulated in most gastric cancer cell lines comparing with GES (Fig. 1a, b) and it’s high expressed in 70.83% (17/24) of gastric cancer patients (Fig. 1c–e). Additionally, we detected the expression of PIN1 by IHC in a tissue chip containing 66 pairs of gastric cancer and adjacent tissues. Then, analyzed the correlation of PIN1 expression with clinical feature. The result also indicated that PIN1 is high expressed in majority of gastric cancer tissues (44/66; Fig. 1f, g) and positive related with lymph node metastasis and TNM stage (Fig. 1h, i). The area under the receiver operating characteristic (ROC) curve (AUC) based on PIN1 expression is 0.7609 (Fig. 1j), suggesting that PIN1 is a potential marker to predict patients’ survival. The higher PIN1 expression indicates poorer prognosis (Fig. 1k). Besides, we detected the PIN1 expression in a tissue chip containing metastasis and corresponding primary lesions. The result shows that PIN1 expression is much higher in the metastasis lesions (Fig. 1l, m). These data confirmed the high expression of PIN1 in gastric cancer and indicated its important role in gastric cancer metastasis.

### PIN1 facilitates the progression of gastric cancer

Recent researches demonstrated that PIN1 promotes the proliferation, colony formation, and chemoresistance of gastric cancer. It’s reported that PIN1 promotes the migration and invasion of glioblastoma^22^, prostate^23^, hepatocellular carcinoma^24^, but gastric cancer. Accordingly, we project to detected the function of PIN1 on gastric cancer metastasis.

**Fig. 2:**
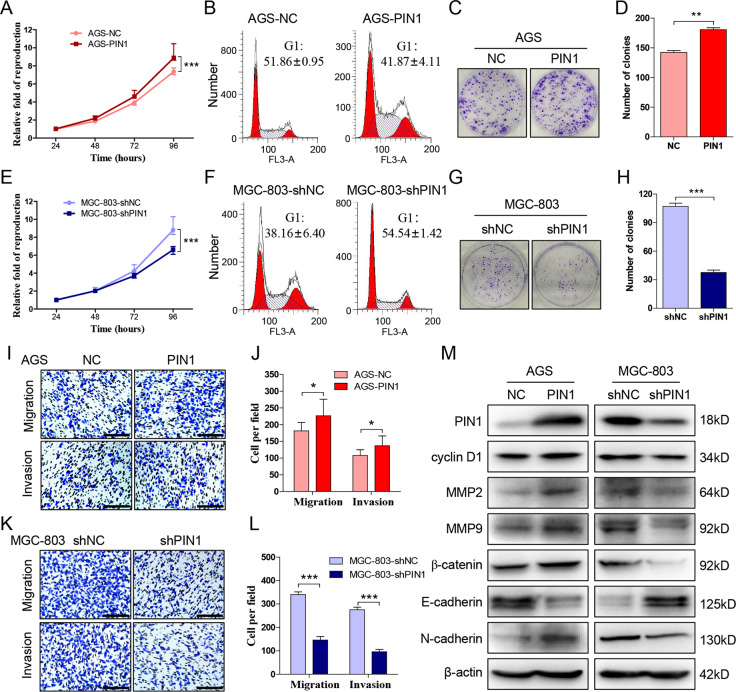
**PIN1 facilitates the progression of gastric cancer.** **a** Detect the proliferation of AGS that exogenous expressed PIN1 or negative control by CCK-8. **b** The cell cycle of cells in **a** are detected by flow cytometry. **c**, **d** The colony formation of cells in **a** and the clone number was counted. **e** Detect the proliferation of MGC-803 that interfered PIN1 or not by CCK-8. **f** The cell cycle of cells in **e** are detected by flow cytometry. **g**, **h** The colony formation of cells in **e** and the clone number was counted. **i**, **j** Transwell test to detect the migration and invasion of treated AGS. The migrated and invaded cells were counted. **k**, **l** Transwell test to detect the migration and invasion of treated MGC-803. The migrated and invaded cells were counted. **m** The protein expression of PIN1 downstreams after intervened PIN1 in AGS and MGC-803. The data are shown as the mean ± s.d. (*n* = 3) in cell lines. *p < 0.05, **p < 0.01, ***p < 0.001. Scale bars: 200 μm.

The previously study have revealed that PIN1 expression is relatively lower in AGS and SGC-7901, therefore, we exogenously expressed PIN1 in these two cell lines. As expect, overexpression of PIN1 not only increases the proliferation (Fig. 2a, Supplementary Fig. 1a) and reduces G1 phase (Fig. 2b, Supplementary Fig. 1b) of AGS and SGC-7901 compared with control groups, but also promotes their colony formation (Fig. 2c, d, Supplementary Fig. 1c, d), migration, and invasion (Fig. 2i, j, Supplementary Fig. 1i, j). Besides, we suppressed PIN1 expression by shRNA in HGC-27 and MGC-803 that showed higher PIN1 expression. Result indicated that depletion of PIN1 inhibits the progression (Fig. 2e, g, h, k, l, Supplementary Fig. 1e, g, h, k, l), and increases the G1 phase arrest (Fig. 2f, Supplementary Fig. 1f) of HGC-27 and MGC-803 significantly.

Additionally, after intervening PIN1 expression, we detected the reported PIN1 targeting genes that related to cancer progression. The result demonstrated that PIN1 significantly promotes the expression of cyclin D1, MMP2, MMP9, β-catenin, and N-cadherin, while inhibits E-cadherin expression in gastric cancer (Fig. 2m, Supplementary Fig. 1m). These data revealed that PIN1 contributes to the the proliferation, colony formation, migration, and invasion of gastric cancer.

### MiR-628-5p suppresses the expression of PIN1 in gastric cancer

**Fig. 3:**
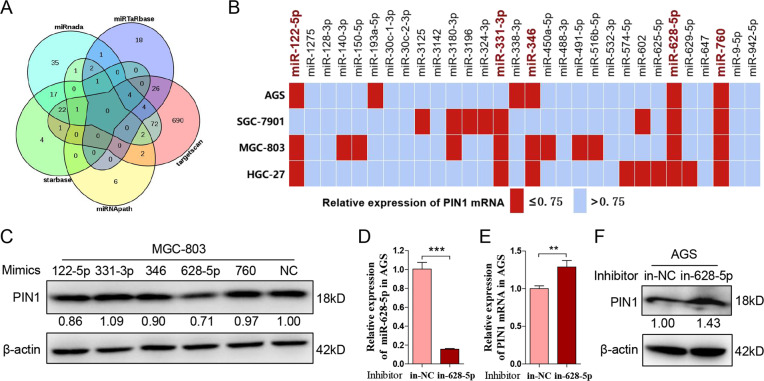
**MiR-628-5p suppresses the expression of PIN1 in gastric cancer.** **a** The prediction of potential PIN1-targeted miRs by miRTarBase, TargetScan, miRNApath, starBase, and miRanda. **b** Four gastric cancer cell lines were transfected different microRNA mimics or negative control, and then PIN1 mRNA was detected. **c** The protein expression of PIN1 in MGC-803 that tranfected different mimics or negative control. The relative protein densitometry was analyzed. **d** The expression of miR-628-5p in AGS that transfected miR-628-5p inhibitor or negative control. **e**, **f** The PIN1 mRNA and protein expression in the treated AGS. The relative protein densitometry was analyzed. The data are shown as the mean ± s.d. (*n* = 3) in cell lines. **p < 0.01, ***p < 0.001.

Researches have revealed that miR-200b-3p, miR-200c-3p, miR-296-5p, miR-874-3p, miR-370, and miR-140-5p target PIN1 in other cancers. To investigate the PIN1-targeted miR in gastric cancer, we predicted miRs that potentially target PIN1 by mirTarbase, targetscan, miRNApath, starbase, and miRanda (Fig. 3a). Thirty-six PIN1-targeted candidates are predicted by at least three databases (Supplementary Table 3). Excepting the six miRs that have confirmed to target PIN1 (Supplementary Table 4), we synthesized mimics of the remained 30 miRs. Each mimic was transfected into four gastric cancer cell lines mentioned above, and the mRNA level of PIN1 was detected at 48 h post transfection. As shown in Fig. 3b, miR-122-5p, miR-331-3p, miR-346, miR-628-5p, and miR-760 reduced the mRNA expression of PIN1 by at least 25% in three or more gastric cancer cell lines. Then, we verified the function of these five miR mimics on the PIN1 protein level. Result revealed that miR-628-5p inhibits the protein expression of PIN1 significantly both in HGC-27 and MGC-803 (Fig. 3c, Supplementary Fig. 2a–c). The further investigation indicated that miR-628-5p inhibitor promotes the expression of PIN1 in AGS and SGC-7901 (Fig. 3d–f, Supplementary Fig. 2d–f). These data revealed that miR-628-5p suppresses PIN1 in gastric cancer.

### MiR-628-5p directly binds to the 3′UTR of PIN1 mRNA

**Fig. 4:**
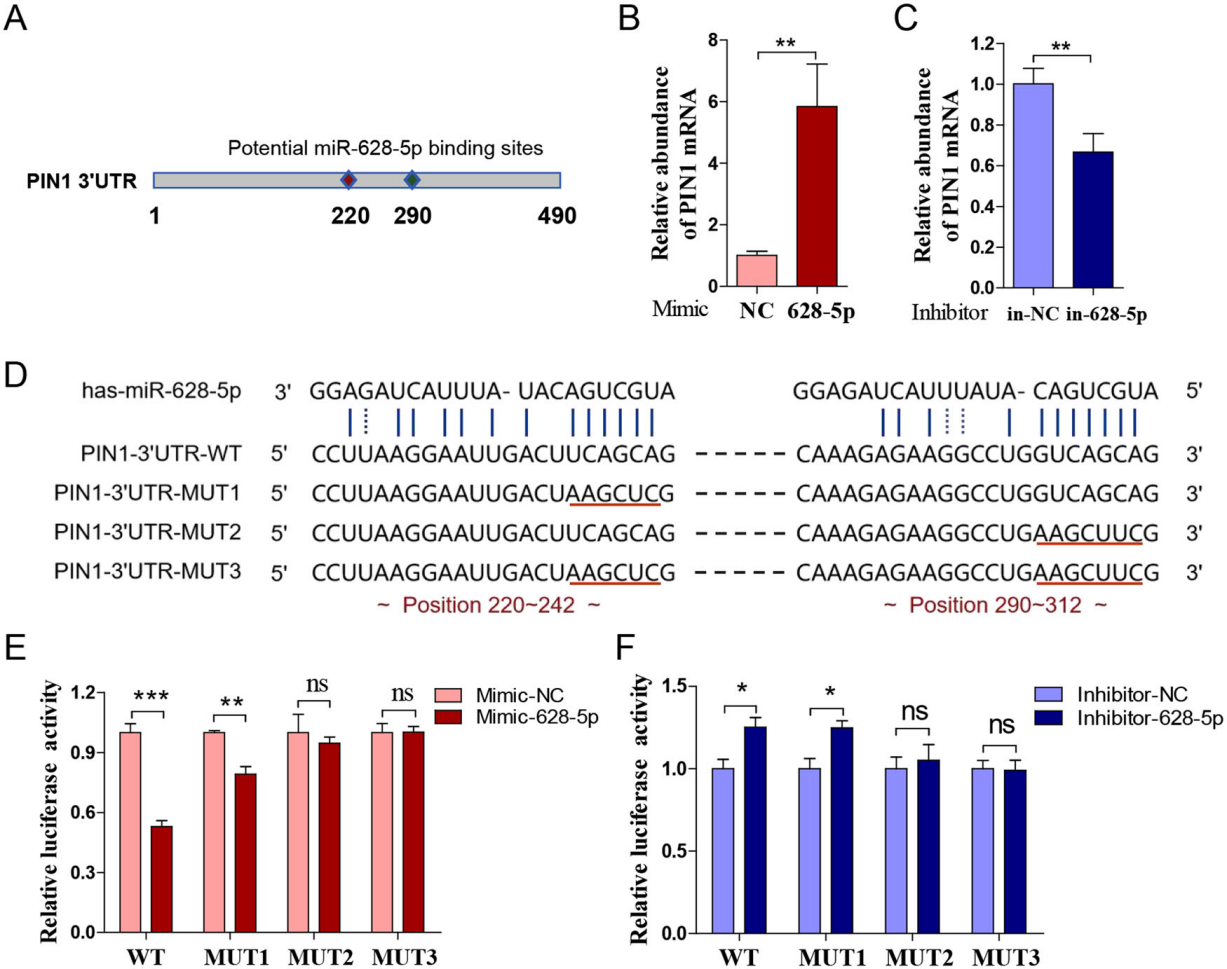
**MiR-628-5p directly binds to the 3′UTR of PIN1 mRNA.** **a** The potential binding sites of miR-6285p in the PIN1 mRNA 3′UTR. **b**, **c** The mRNA abundance of PIN1 mRNA in AGO2 immunoprecipitates that from SGC-7901-transfected miR-628-5p mimic, inhibitor, or their negative control. **d** The sequence of the WT or mutant PIN1 3′UTR reporter plasmid. **e**, **f** The relative luciferase activity in SGC-7901 that transfected miR-628-5p mimic, inhibitor, or their corresponding negative control that cotransfected with WT or mutant PIN1 3′UTR reporter plasmid. The data are shown as the mean ± s.d. (*n* = 3) in cell lines. *p < 0.05, **p < 0.01, ***p < 0.001, ns, no significance.

Subsequently, we determined to make it certain whether miR-628-5p is a novel PIN1-targeted miR. miRs generally bind to the 3′UTR of their target genes. According to the prediction, there are two potential miR-628-5p binding sites (220 and 290) in the 3′UTR of PIN1 mRNA (Fig. 4a). RIP assay was applied to testify whether miR-628-5p bind to the 3′UTR of PIN1 mRNA specifically. After transfecting miR-628-5p mimic into MGC-803, the abundance of PIN1 mRNA increased significantly in the AGO2 immunoprecipitates (Fig. 4b). Meanwhile, the abundance of PIN1 mRNA in the AGO2 immunoprecipitates from AGS-transfected miR-628-5p inhibitor decreased significantly (Fig. 4c). Additionally, we purchased PIN1 3′UTR luciferase reporter plasmid from Sangon Biotech and constructed three different mutant that renamed as pPIN1-3′UTR-WT, pPIN1-3′UTR-MUT1, pPIN1-3′UTR-MUT2, and pPIN1-3′UTR-MUT3, respectively (Fig. 4d). The result showed that cotransfection of miR-628-5p mimic in SGC-7901 reduces the relatively luciferase activity of pPIN1-3′UTR-WT and pPIN1-3′UTR-MUT1 significantly, but not pPIN1-3′UTR-MUT2 and pPIN1-3′UTR-MUT3 (Fig. 4e). Meanwhile, cotransfection of miR-628-5p inhibitor only increases the relatively luciferase activity of pPIN1-3′UTR-WT and pPIN1-3′UTR-MUT1 (Fig. 4f). These data indicated that miR-628-5p directly binds to the 290 site of PIN1 mRNA 3′UTR to inhibit the expression of PIN1.

### MiR-628-5p suppresses the progression of gastric cancer

**Fig. 5:**
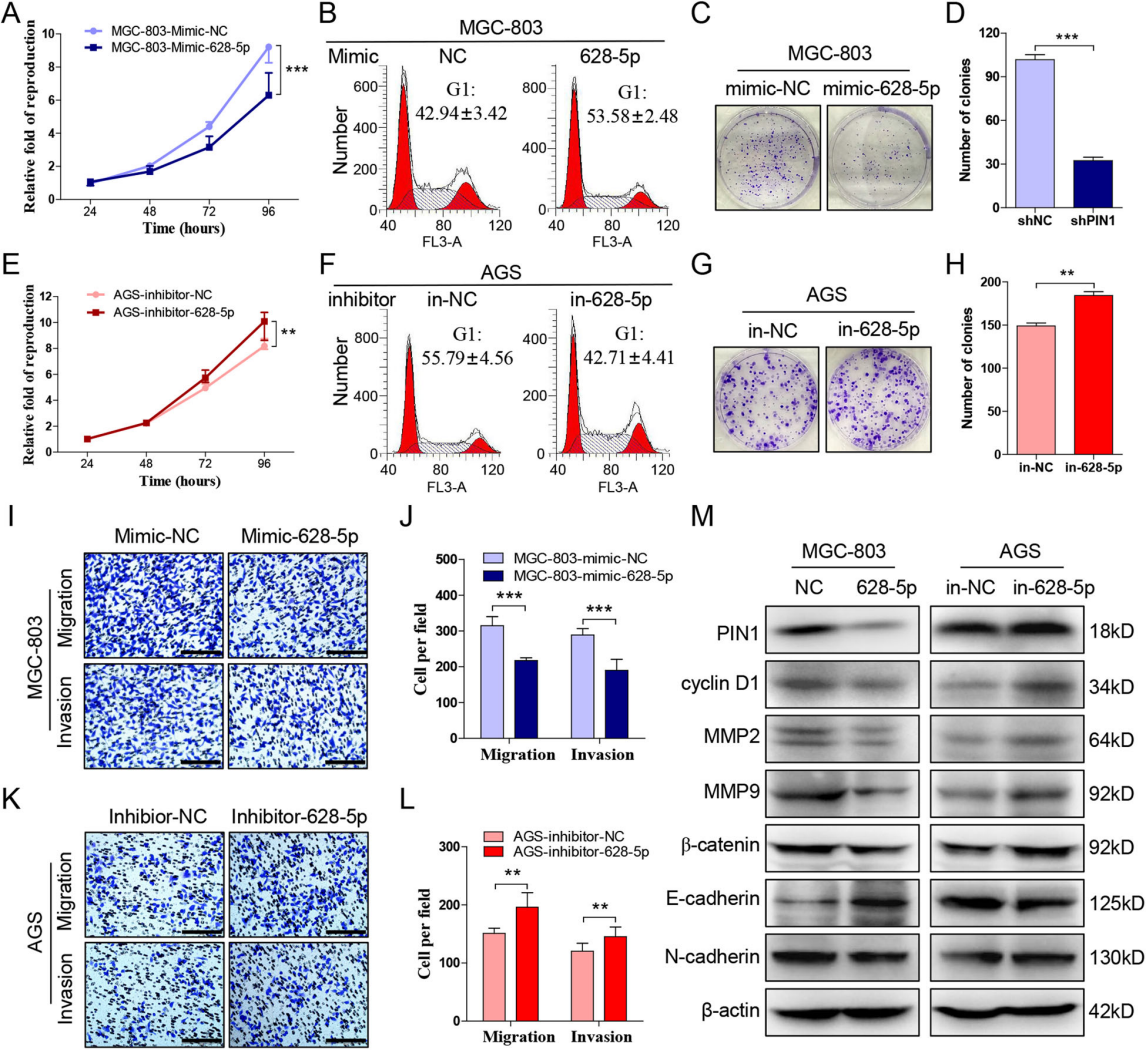
**MiR-628-5p suppresses the progression of gastric cancer.** **a** CCK-8 to detect the proliferation of MGC-803 that transfected miR-628-p mimic or negative control. **b** The cell cycle of cells in **a** are detected by flow cytometry. **c**, **d** The colony formation of cells in **a** and the clone number was counted. **e** Detect the proliferation of AGS that transfected miR-628-p inhibitor or negative control. **f** The cell cycle of cells in **e** are detected by flow cytometry. **g**, **h** The colony formation of cells in **e** and the clone number was counted. **i**, **j** Transwell test to detect the migration and invasion of treated HGC-27. The migrated and invaded cells were counted. **k**, **l** Transwell test to detect the migration and invasion of treated AGS. The migrated and invaded cells were counted. **m** The protein expression of PIN1 downstreams after intervened miR-628-5p in MGC-803 and AGS. The data are shown as the mean ± s.d. (*n* = 3) in cell lines. **p < 0.01, ***p < 0.001. Scale bars: 200 μm.

Researches indicated that miR-628-5p suppresses multiple cancers, including prostate cancer^19^, glioma^20^, and epithelial ovarian cancer^21^. Accordingly, we transfected the miR-628-5p mimic into MGC-803 and HGC-27, and the result showed that their proliferation (Fig. 5a, Supplementary Fig. 3a), colony formation (Fig. 5c, d, Supplementary Fig. 3c, d), migration, and invasion (Fig. 5i, j, Supplementary Fig. 3i, j) were suppressed while G1 arrest were increased significantly (Fig. 5b, Supplementary Fig. 3b). Meanwhile, inhibiting miR-628-5p in AGS and SGC-7901 promotes their proliferation (Fig. 5e, Supplementary Fig. 3e), colony formation (Fig. 5g, h, Supplementary Fig. 3g, h), migration, invasion (Fig. 5k, l, Supplementary Fig. 3k, l), and reversed the G1 arrest (Fig. 5f, Supplementary Fig. 3f). Similarly, we detected the PIN1 downstream genes mentioned above and the result showed that miR-628-5p decreases the expression of cyclin D1, MMP2, MMP9, β-catenin, N-cadherin, and promotes E-cadherin expression (Fig. 5m, Supplementary Fig. 3m). These data demonstrated that miR-628-5p also inhibits gastric cancer.

### PIN1 reverses the miR-628-5p-mediated suppression of gastric cancer

**Fig. 6:**
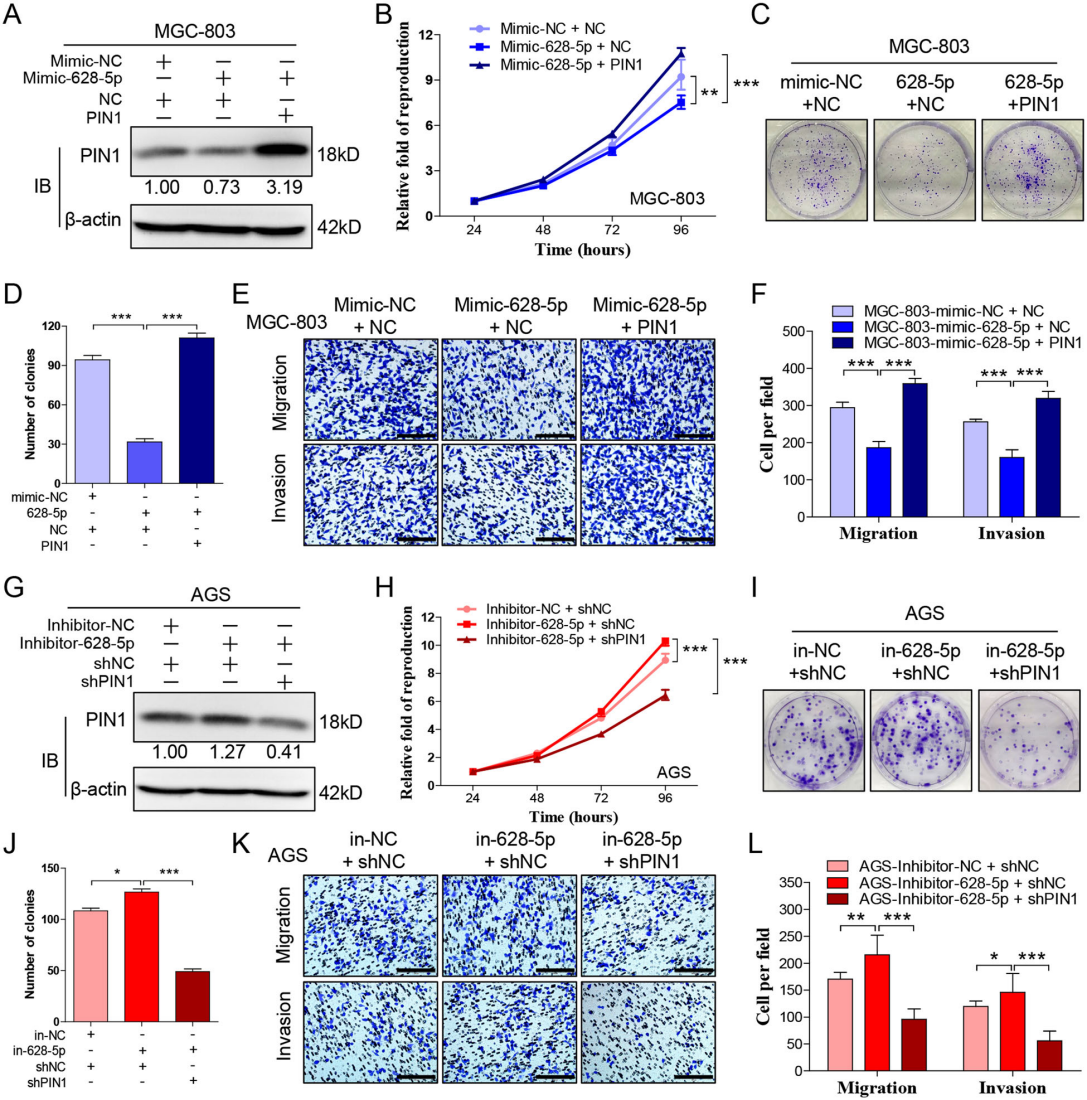
**PIN1 reverses the miR-628-5p-mediated suppression of gastric cancer.** **a** The protein expression of PIN1 in MGC-803 that cotransfected miR-628-5p mimic and PIN1 overexpression plasmid. The relative protein densitometry was analyzed. **b** CCK-8 to detect the proliferation of cells in **a**. **c**, **d** The colony formation of cells in **a** and the clone number was counted. **e**, **f** Transwell test to detect the migration and invasion of cells in **a**. The migrated and invaded cells were counted. **g** The protein expression of PIN1 in AGS that cotransfected miR-628-5p inhibitor and PIN1 shRNA plasmid. The relative protein densitometry was analyzed. **h** CCK-8 to detect the proliferation of cells in **g**. **i**, **j** The colony formation of cells in **g** and the clone number was counted. **k**, **l** Transwell test to detect the migration and invasion of cells in **g**. The migrated and invaded cells were counted. The data are shown as the mean ± s.d. (*n* = 3) in cell lines. *p < 0.05, **p < 0.01, ***p < 0.001. Scale bars: 200 μm.

Research above has indicated that miR-628-5p suppresses the expression of PIN1 and progression of gastric cancer. Our following research aimed to determine whether miR-628-5p suppresses gastric cancer through inhibiting PIN1. Accordingly, we recovered the expression of PIN1 in HGC-27 or MGC-803 that transfected miR-628-5p mimic. Result showed that, overexpression of PIN1 reverses the miR-628-5p-mediated suppression of gastric cancer (Fig. 6a–f, Supplementary Fig. 4a–f). Additionally, we interfered PIN1 in AGS or SGC-7901 that transfected miR-628-5p inhibitor. Similarly, inhibiting PIN1 counteracts the cancer-promoting effect of miR-628-5p inhibitor completely (Fig. 6g–l, Supplementary Fig. 4g–l). These data demonstrated that miR-628-5p can inhibit gastric cancer through inhibiting PIN1.

### MiR-628-5p is downregulated in gastric cancer tissue especially metastasis tissue

**Fig. 7:**
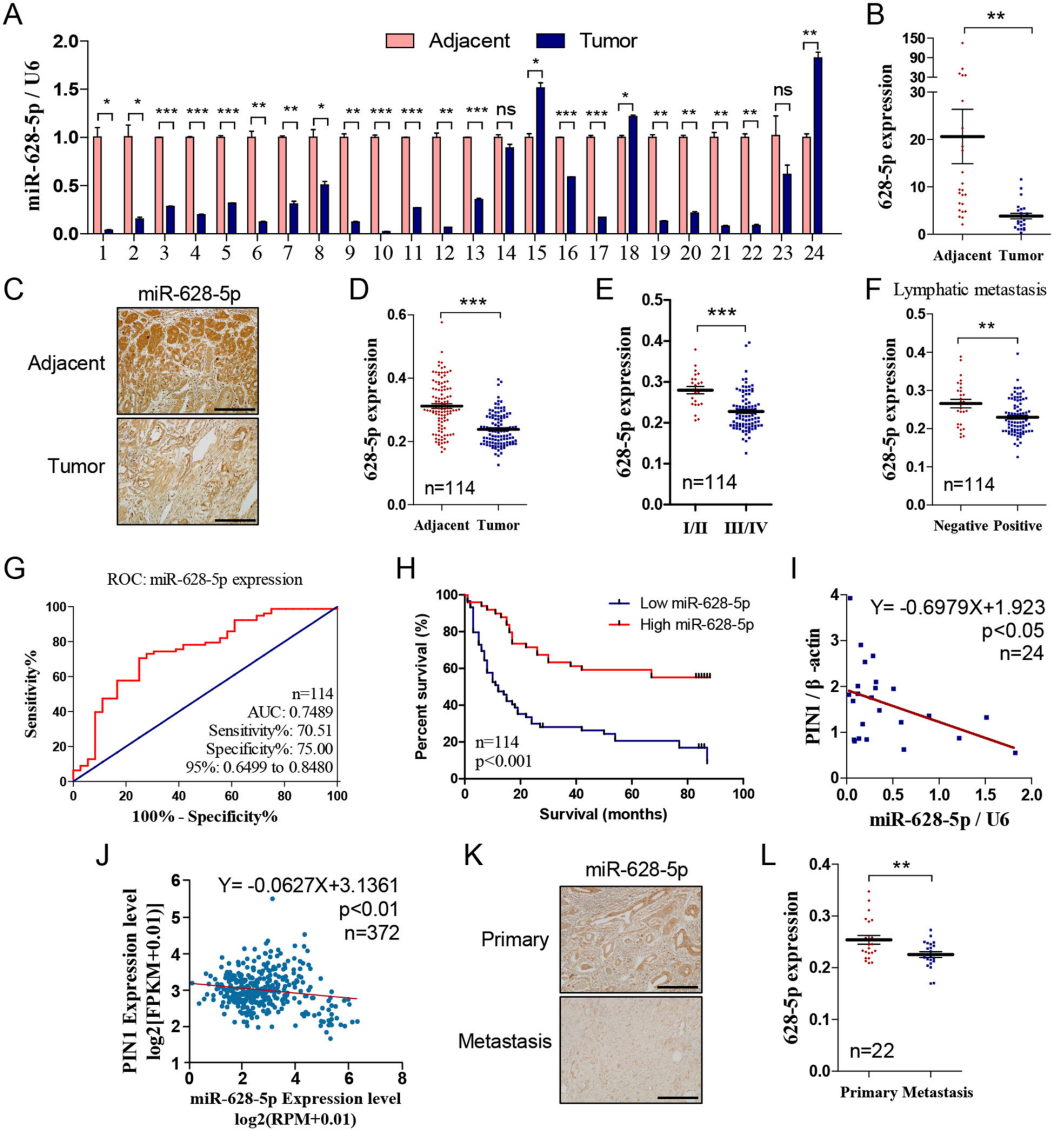
**MiR-628-5p is downregulated in gastric cancer tissue especially metastasis tissue.** **a**, **b** The miR-628-5p expression in the 24 pairs of cancer and adjacent tissue (*n* = 24). **c** Representative in situ hybridization staining of miR-628-5p in the cancer and adjacent tissue. **d** The miR-628-5p expression in the cancer and adjacent tissue (*n* = 114). **e**, **f** The correlation of miR-628-5p expression with TNM stage/lymphatic metastasis in gastric cancer (*n* = 114). **g** The ROC curves based on miR-628-5p expression to predict patients’ survival time. **h** The miR-628-5p expression based overall survival of gastric cancer patient by Kaplan–Meier analyses (*n* = 114, *p* < 0.01). **i**, **j** The correlation of miR-628-5p and PIN1 expression in the 24 gastric cancer tissues and TCGA project. **k** Representative in situ hybridization staining of miR-628-5p in the primary and metastasis tissue. **l** The miR-628-5p expression in the primary and corresponding metastasis cancer tissues (*n* = 22). The data are shown separately in human samples, **p < 0.01, ***p < 0.001. Scale bars: 200 μm.

The expression of miR-628-5p is lower in prostate cancer^19^, glioma^25^, and epithelial ovarian cancer^21^. To determine whether high expression of PIN1 is related to deficiency of miR-628-5p in gastric cancer, we detected the expression of miR-628-5p in the 24 pairs of gastric cancer and adjacent tissues by qRT-PCR. Result revealed that miR-628-5p expression is insufficient in 87.5% (21/24) cases (Fig. 7a, b). Besides, we detected miR-628-5p expression by ISH in tissue chip containing 114 pairs of cancer and adjacent tissues. The result confirmed that miR-628-5p is downregulated in most gastric cancer (91/114; Fig. 7c, d), and also revealed that miR-628-5p expression is negative correlated with TNM stage and lymphatic metastasis (Fig. 7e, f). The AUC based on miR-628-5p expression is 0.7489 (Fig. 7g) and the lower expression of miR-628-5p indicates poorer survival rate (Fig. 7h), indicating that the miR-628-5p level could be a potential prediction of gastric cancer patient’s prognosis. Meanwhile, miR-628-5p and PIN1 expression were negatively correlated in the 24 clinic patients (Fig. 7i) and data from TCGA project containing 372 patients (Fig. 7j, the miR-628-5p and PIN1 expression data were scaled with log2(RPM + 0.01) and log2(FPKM + 0.01), respectively, the figure was quoted from starbase.sysu.edu.cn). Additionally, we detected the expression of miR-628-5p in a gastric cancer tissue chip containing primary and metastasis tissues. The result indicated that miR-628-5p expression is significantly lower in the metastasis lesion compared with its primary site (Fig. 7k, l).

In summary, our research indicated that miR-628-5p is a novel miR that suppress gastric cancer by targeting PIN1 directly. The deficiency of miR-628-5p attenuates its inhibition on PIN1 expression and following gastric cancer progresses.

## Discussion

Ser/Thr-Pro motif is abundant in proteins and the Pro-directed Ser/Thr kinase phosphorylates these sites to regulate multiple cellular pathways^26^. PIN1 uniquely catalyzes the conformational transformation of pSer/Thr-Pro motif to modify these pathways, therefore, PIN1 is vital in the normal physiological processes. The disordered PIN1 expression and/or activity is related to various diseases, such as Alzheimer’s disease and cancer. Researches indicated that PIN1 is upregulated in cancer and indicates poor clinical prognosis. It facilitates the progression, including proliferation, migration, invasion, and chemoresistance, of multiple cancers.

The expression of PIN1 is regulated by many factors at different level. E2F family binds to the PIN1 promoter directly to increase its expression^27^. PIN1 suppresses retinoblastoma protein (pRb) to activate E2F that form a positive regulation loop^28,29^. The similar regulation loop also appeared in NOTCH-mediated high expression of PIN1 (ref. ^30^). miR-200b-3p, miR-200c-3p, miR-296-5p, miR-874-3p, miR-370, and miR-140-5p, which were always downregulated in cancers, have demonstrated to inhibit PIN1 expression. PIN1 prevents miR maturation by inhibiting the nucleus-to-cytoplasm transport of exportin-5 (XPO5)^31^, therefore, the PIN1-miR regulation loop require further investigation. Interestingly, we found that PIN1 also inhibits the cytoplasm location of XPO5 in gastric cancer but it has tiny influence on the miR-628-5p expression (Supplementary Fig. 5a, b), indicating that other exportin protein may involved in pre-miR transport. Furthermore, the posttranslational modification also regulates the activity and stability of PIN1 (refs. ^32–34^).

Recently, researches indicated that PIN1 is also upregulated in gastric cancer, and facilitates its proliferation^9,10^ and chemoresistance^11^. Metastasis is one of the leading cause of poor prognosis in gastric cancer^35^. In this study, we revealed that PIN1 not only increases the proliferation and colony formation of gastric cancer, but also promotes its invasion and migration. The PIN1 expression is usually higher in the metastasis lesion compared with the corresponding primary site. Researches have indicated that miR-628-5p inhibits prostate cancer, glioma, epithelial ovarian cancer by targeting different downstreams. We firstly demonstrated that miR-628-5p also inhibits the progression of gastric cancer through targeting PIN1 at the 290 region of its mRNA 3′UTR. However, as in other cancers, the expression of miR-628-5p is deficient in majority of gastric cancer tissues especially in metastasis tissues that contributes to the high expression of PIN1 and facilitates the progresses of gastric cancer. Additionally, serum level of miR-628-5p has reported to be an indicator of preeclampsia^36^, cardiac allograft vasculopathy^37^, and prostate cancer^38^. Hence, it is meaningful to confirm whether circulatory miR-628-5p could be a universal biomarker of different cancers.

Many research have indicated that PIN1 inhibitors suppress different cancers in cell and animal test significantly^24,39^; however, it is far to apply in clinic treatment for the vital role of PIN1 in normal physiological progresses and the specificity of PIN1 inhibitors. RNA interference (RNAi) is effective and specific to silence targeted gene. Many RNAi-based therapeutic are in the clinical research stage and even one of them was approved by United States Food and Drug Administration^40^. The PIN1-targeted RNAi has been proved to suppress prostate cancer in vitro and mice model^41^. Our study provided a potential target for the RNAi-based therapeutic by inhibiting PIN1 in gastric cancer. However, due to the tumor heterogeneity, whether miR-628-5p targets PIN1 in other cancers and the circulatory miR-628-5p level acting as an indicator in different cancers require further investigation.

## Supplementary information

Supplementary information

Supplementary information 2

Supplementary information 3

Supplementary information 4

Supplementary information 5

Supplementary information 6

Supplementary information 7

Supplementary information 8

Supplementary information 9

Supplementary information 10
