# Supplementary material for: Deficiency of microRNA-628-5p promotes the progression of gastric cancer by upregulating PIN1
Source: Cell Death Dis. 2020 Jul 23;11(7):559. doi: 10.1038/s41419-020-02766-6 (PMC7378826; doi:10.1038/s41419-020-02766-6)
Supplement: Supplementary file 1 — Supplementary information [file 41419_2020_2766_MOESM1_ESM.docx]

**Figure S1. PIN1 facilitates the progression of gastric cancer.**

1. Detect the proliferation of SGC-7901 that exogenous expressed PIN1 or negative control by CCK-8.

(B) The cell cycle of cells in A are detected by flow cytometry.

(C,D) The colony formation of cells in A and the clone number was counted.

(E) Detect the proliferation of HGC-27 that interfered PIN1 or not by CCK-8.

(F) The cell cycle of cells in E are detected by flow cytometry.

(G,H) The colony formation of cells in E and the clone number was counted.

(I,J) Transwell test to detect the migration and invasion of treated SGC-7901. The migrated and invaded cells were counted.

(K,L) Transwell test to detect the migration and invasion of treated HGC-27. The migrated and invaded cells were counted.

(M) The protein expression of PIN1 downstreams after intervened PIN1 in SGC-7901 and HGC-27.

The data are shown as the mean ± s.d. (n = 3) in cell lines. **p<0.01, ***p<0.001. Scale bars : 200μm.

**Figure S2. MiR-628-5p suppresses the expression of PIN1 in gastric cancer.**

(A,B) The expression of miRs in MGC-803 or HGC-27 that transfected corresponding mimic or negative control.

(C) The protein expression of PIN1 in HGC-27 that tranfected different mimic or negative control. The relative protein densitometry was analyzed.

(D) The expression of miR-628-5p in SGC-7901 that transfected miR-628-5p inhibitor or negative control.

(E,F) The mRNA and protein expression of PIN1 in the treated SGC-7901. The relative protein densitometry was analyzed.

The data are shown as the mean ± s.d. (n = 3) in cell lines. *p < 0.05, **p<0.01, ***p<0.001.

**Figure S3. MiR-628-5p suppresses the progression of gastric cancer.**

1. CCK-8 to detect the proliferation of HGC-27 that transfected miR628-p mimic or negative control.
2. The cell cycle of cells in A are detected by flow cytometry.

(C,D) The colony formation of cells in A and the clone number was counted.

(E) Detect the proliferation of SGC-7901 that transfected miR628-5p inhibitor or negative control.

(F) The cell cycle of cells in E are detected by flow cytometry.

(G,H) The colony formation of cells in E and the clone number was counted.

(I,J) Transwell test to detect the migration and invasion of treated HGC-27. The migrated and invaded cells were counted.

(K,L) Transwell test to detect the migration and invasion of treated SGC-7901. The migrated and invaded cells were counted.

(M) The protein expression of PIN1 downstreams after intervened miR-628-5p in HGC-27 and SGC-7901.

The data are shown as the mean ± s.d. (n = 3) in cell lines. *p < 0.05, **p<0.01, ***p<0.001. Scale bars : 200μm.

**Figure S4. PIN1 reverses the miR-628-5p-mediated suppression of gastric cancer.**

1. The protein expression of PIN1 in HGC-27 that cotransfected miR-628-5p mimic and PIN1 overexpression plasmid. The relative protein densitometry was analyzed.

(B) CCK-8 to detect the proliferation of cells in A.

(C-D) The colony formation of cells in A and the clone number was counted.

(E,F) Transwell test to detect the migration and invasion of cells in A. The migrated and invaded cells were counted.

(G) The protein expression of PIN1 in SGC-7901 that cotransfected miR-628-5p inhibitor and PIN1 shRNA plasmid. The relative protein densitometry was analyzed.

(H) CCK-8 to detect the proliferation of cells in G.

(I,J) The colony formation of cells in G and the clone number was counted.

(K,L) Transwell test to detect the migration and invasion of cells in G. The migrated and invaded cells were counted.

The data are shown as the mean ± s.d. (n = 3) in cell lines. *p < 0.05, **p<0.01, ***p<0.001. Scale bars : 200μm.

**Figure S5. PIN1 don’t inhibit the maturation of miR-628-5p in gastric cancer.**

(A) The total and nucleal level of XPO5 protein after intervening PIN1.

(B) The miR-628-5p expression after intervening PIN1. The data are shown as the mean ± s.d. (n = 3) in cell lines, *p<0.05, ns, no significance.
