# Supplementary figures and images for: Deficiency of microRNA-628-5p promotes the progression of gastric cancer by upregulating PIN1
Source: Cell Death Dis. 2020 Jul 23;11(7):559. doi: 10.1038/s41419-020-02766-6 (PMC7378826; doi:10.1038/s41419-020-02766-6)

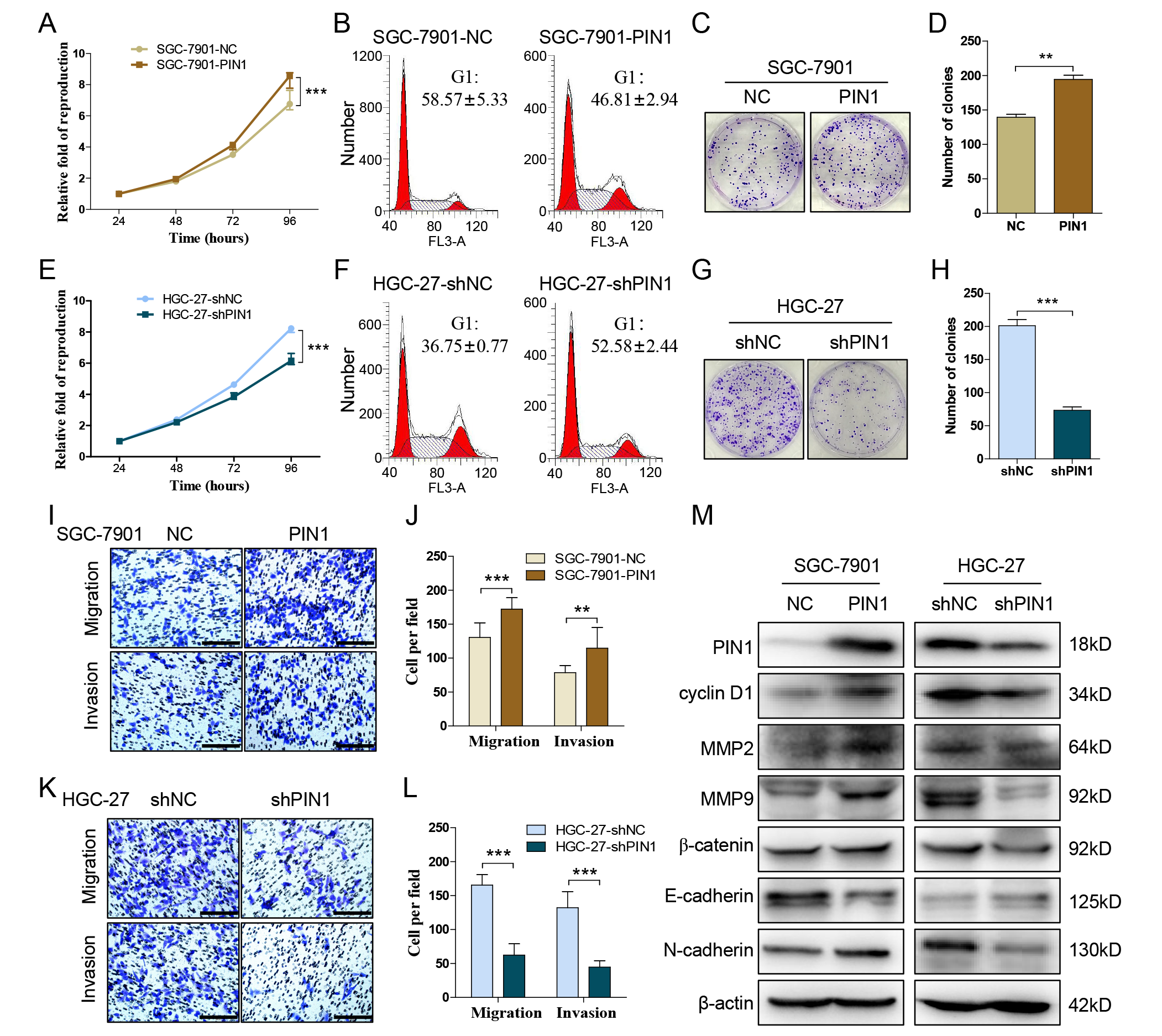

Supplement: Supplementary file 2 — Supplementary information 2 [file 41419_2020_2766_MOESM2_ESM.tif]

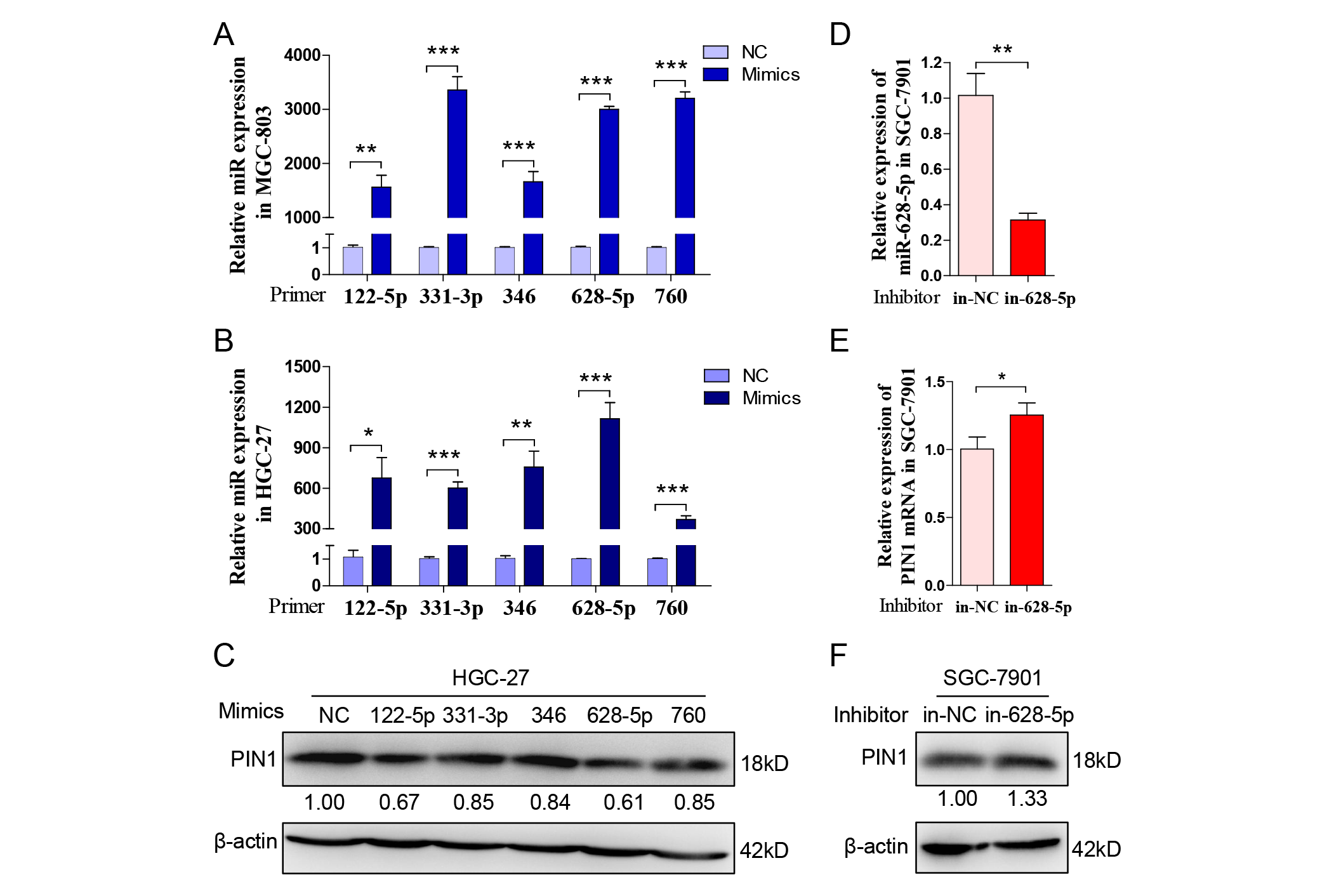

Supplement: Supplementary file 3 — Supplementary information 3 [file 41419_2020_2766_MOESM3_ESM.tif]

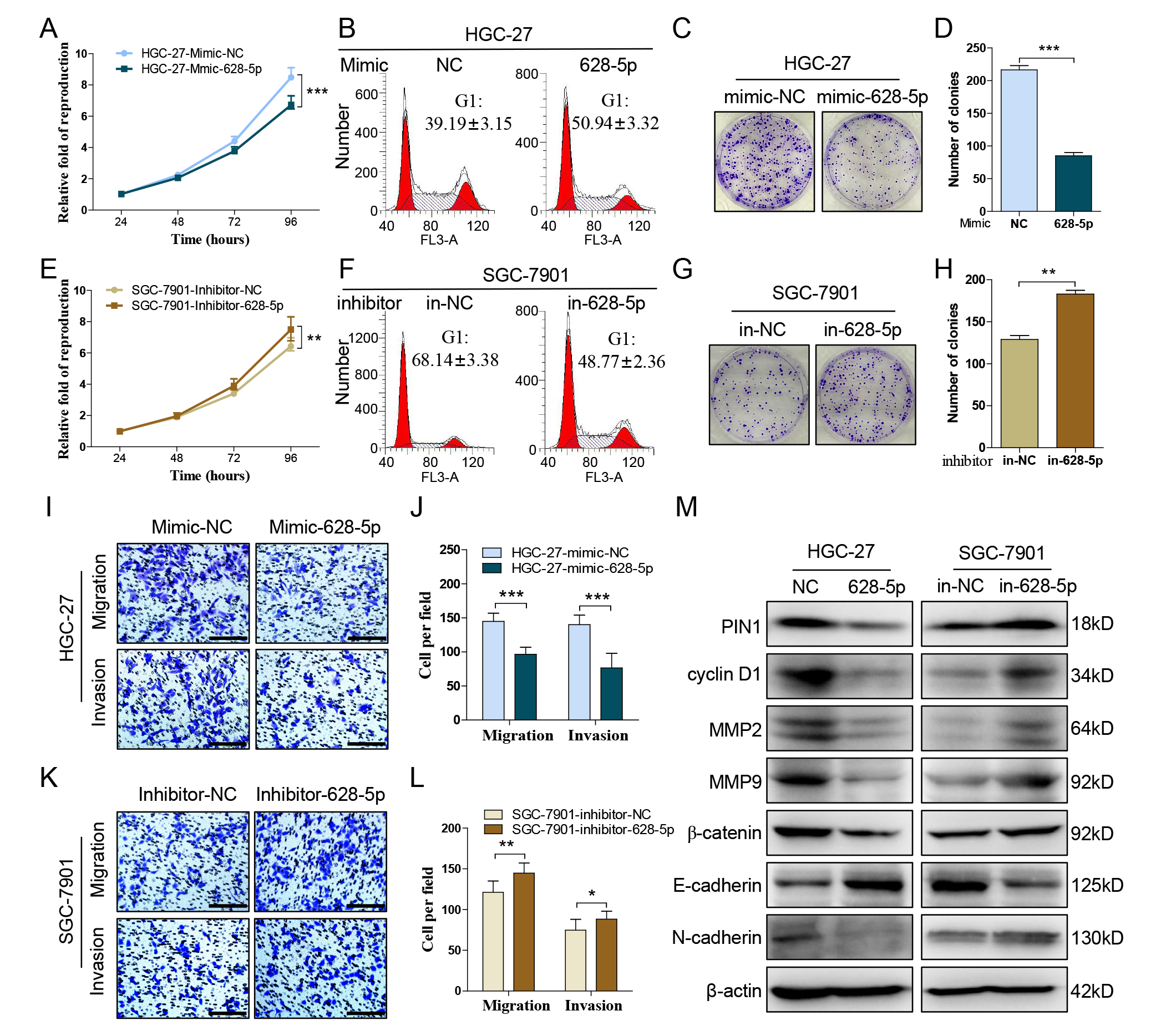

Supplement: Supplementary file 4 — Supplementary information 4 [file 41419_2020_2766_MOESM4_ESM.tif]

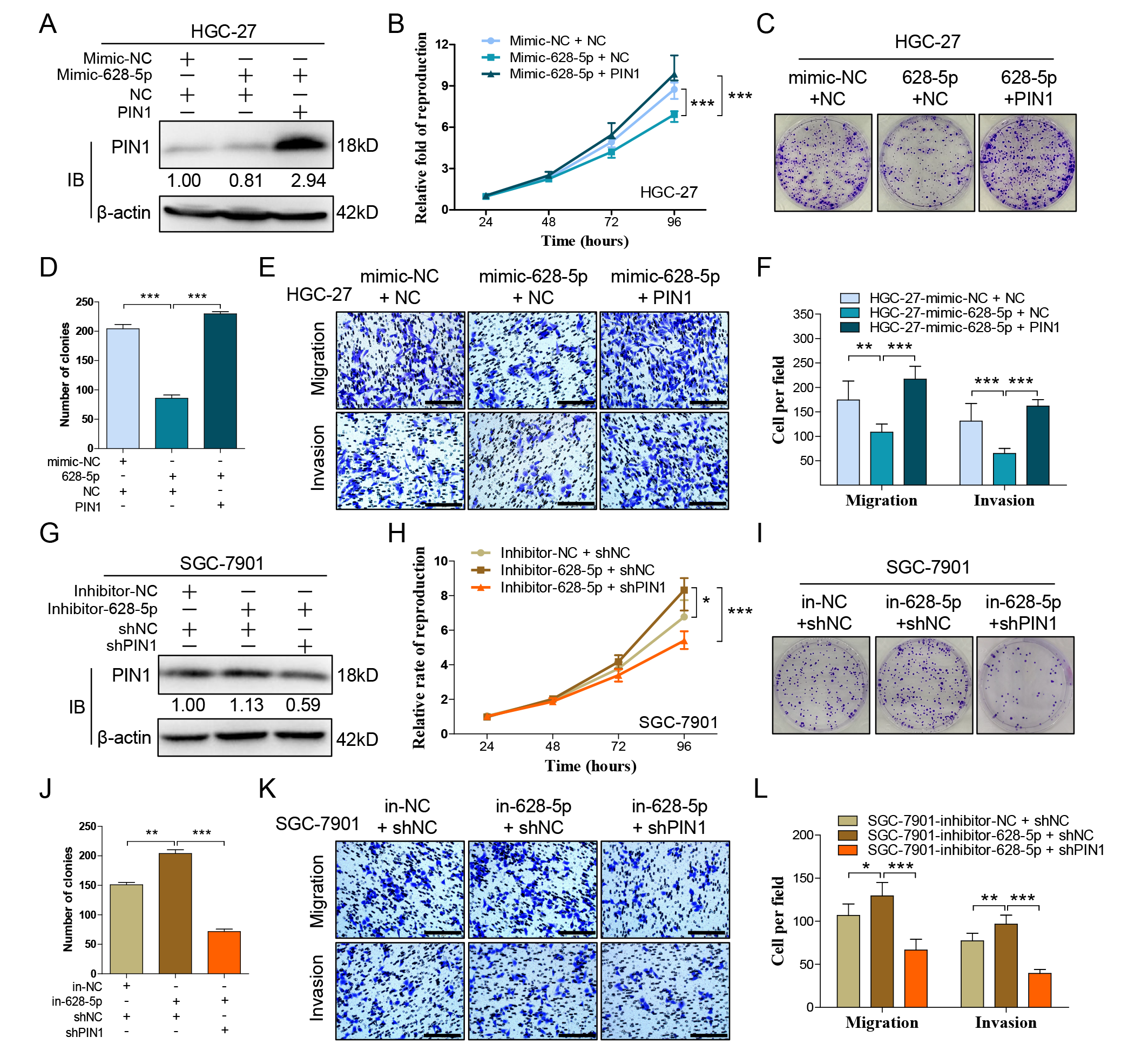

Supplement: Supplementary file 5 — Supplementary information 5 [file 41419_2020_2766_MOESM5_ESM.tif]

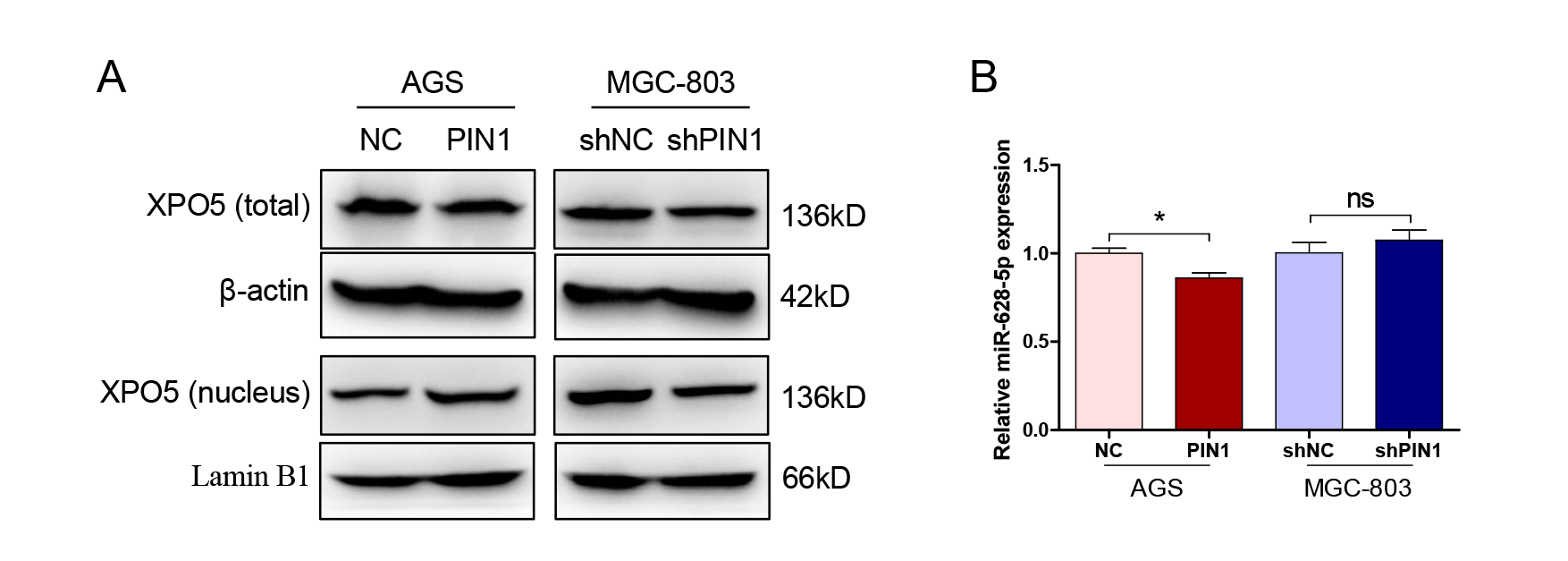

Supplement: Supplementary file 6 — Supplementary information 6 [file 41419_2020_2766_MOESM6_ESM.tif]
