# Supplementary material for: Deficiency of microRNA-628-5p promotes the progression of gastric cancer by upregulating PIN1
Source: Cell Death Dis. 2020 Jul 23;11(7):559. doi: 10.1038/s41419-020-02766-6 (PMC7378826; doi:10.1038/s41419-020-02766-6)
Supplement: Supplementary file 7 — Supplementary information 7 [file 41419_2020_2766_MOESM7_ESM.doc]

Table S1. Sequence of primers

| **Primer** | **Sequence** | **application** |
| --- | --- | --- |
| PIN1-F | CGGGGTACCGCCACCATGGCGGACGAGGAGAAGCTG | PIN1 CDS  amplification |
| PIN1-R | CCCAAGCTTTCACTCAGTGCGGAGGATGATGTG |
| PIN1-3’UTR-MUT220-F | GACTAAGCTCGGGGTGGGAGGCTCCCAG | Mutant reporter plasmid construction |
| PIN1-3’UTR-MUT220-R | CCCCGAGCTTAGTCAATTCCTTAAGGGAGAATCTG |
| PIN1-3’UTR-MUT290-F | CTGAAGCTTCGAGCCGCCCCGTGTC |
| PIN1-3’UTR-MUT290-R | CTCGAAGCTTCAGGCCTTCTCTTTGGAACACC |
| miR-122-5p-RT | GTCGTATCCAGTGCAGGGTCCGAGGTATTCGCACTGGATACGACCAAACACC | miR and U6 reverse transcription |
| miR-331-3p-RT | GTCGTATCCAGTGCAGGGTCCGAGGTATTCGCACTGGATACGACTTCTAGGA |
| miR-346-RT | GTCGTATCCAGTGCAGGGTCCGAGGTATTCGCACTGGATACGACAGAGGCAG |
| miR-628-5p-RT | GTCGTATCCAGTGCAGGGTCCGAGGTATTCGCACTGGATACGACCCTCTAGT |
| miR-760-RT | GTCGTATCCAGTGCAGGGTCCGAGGTATTCGCACTGGATACGACTCCCCACA |
| U6-RT | AACGCTTCACGAATTTGCGT |
| miR-122-5p-q-F | GACGGGCTGGAGTGTGACAAT | qPCR primer for miR |
| miR-331-3p-q-F | TCCTGCCCCTGGGCCTA |
| miR-346-q-F | CCTGTCTGCCCGCATGC |
| miR-628-5p-q-F | GCGGGCATGCTGACATATTT |
| miR-760-q-F | CAATCTGCGGCTCTGGGTC |
| miR-universal-q-R | GGGTCCGAGGTATTCGCACT |
| U6-q-F | CTCGCTTCGGCAGCACATATA | qPCR primer for U6 |
| U6-q-R | AACGCTTCACGAATTTGCGT |
| PIN1-q-F | TCGCACCTGCTGGTGAA | qPCR primer for PIN1 |
| PIN1-q-R | ACTGTGAGGCCAGAGAC |
| β-actin-q-F | ATCGTGCGTGACATTAAGGAGAAG | qPCR primer for β-actin |
| β-actin-q-R | AGGAAGGAAGGCTGGAAGAGTG |
