# Supplementary material for: Deficiency of microRNA-628-5p promotes the progression of gastric cancer by upregulating PIN1
Source: Cell Death Dis. 2020 Jul 23;11(7):559. doi: 10.1038/s41419-020-02766-6 (PMC7378826; doi:10.1038/s41419-020-02766-6)
Supplement: Supplementary file 8 — Supplementary information 8 [file 41419_2020_2766_MOESM8_ESM.doc]

Table S2. Sequence of PIN1 3’UTR and the potential miR-628-5p binding sites

| **Name** | **Sequence** |
| --- | --- |
| Sequence of the PIN1 3’UTR | GGGTGGGGAGCCCAGGCCTGGCCTCGGGGCAGGGCAGGGCGGCTAGGCCGGCCAGCTCCCCCTTGCCCGCCAGCCAGTGGCCGAACCCCCCACTCCCTGCCACCGTCACACAGTATTTATTGTTCCCACAATGGCTGGGAGGGGGCCCTTCCAGATTGGGGGCCCTGGGGTCCCCACTCCCTGTCCATCCCCAGTTGGGGCTGCGACCGCCAGATTCTCCCTTAAGGAATTGACTTCAGCAGGGGTGGGAGGCTCCCAGACCCAGGGCAGTGTGGTGGGAGGGGTGTTCCAAAGAGAAGGCCTGGTCAGCAGAGCCGCCCCGTGTCCCCCCAGGTGCTGGAGGCAGACTCGAGGGCCGAATTGTTTCTAGTTAGGCCACGCTCCTCTGTTCAGTCGCAAAGGTGAACACTCATGCGGCCCAGCCATGGGCCCTCTGAGCAACTGTGCAGCACCCTTTCACCCCCAATTAAACCCAGAACCACTGCTCTGC |
| Potential miR-628-5p binding site 220 | CCTTAAGGAATTGACTTCAGCAG |
| Potential miR-628-5p binding site 290 | CAAAGAGAAGGCCTGGTCAGCAG |
