# Supplementary material for: Deficiency of microRNA-628-5p promotes the progression of gastric cancer by upregulating PIN1
Source: Cell Death Dis. 2020 Jul 23;11(7):559. doi: 10.1038/s41419-020-02766-6 (PMC7378826; doi:10.1038/s41419-020-02766-6)
Supplement: Supplementary file 9 — Supplementary information 9 [file 41419_2020_2766_MOESM9_ESM.doc]

Table S3. Putative microRNAs targeting PIN1

| NM. | Database | | | | | Sequence |
| --- | --- | --- | --- | --- | --- | --- |
|  | miRTaRbase | targetscan | miRNApath | starbase | miRanda |  |
| 1 |  | hsa-miR-122-5p |  | hsa-miR-122-5p | hsa-miR-122-5p | uggagugugacaaugguguuug |
| 2 | hsa-miR-1275 | hsa-miR-1275 |  |  | hsa-miR-1275 | gugggggagaggcuguc |
| 3 |  | hsa-miR-128-3p |  | hsa-miR-128-3p | hsa-miR-128-3p | ucacagugaaccggucucuuu |
| 4 |  | hsa-miR-140-3p |  | hsa-miR-140-3p | hsa-miR-140-3p | uaccacaggguagaaccacgg |
| 5 | hsa-miR-140-5p |  | hsa-miR-140-5p | hsa-miR-140-5p | hsa-miR-140-5p | cagugguuuuacccuaugguag |
| 6 |  | hsa-miR-150-5p |  | hsa-miR-150-5p | hsa-miR-150-5p | ucucccaacccuuguaccagug |
| 7 |  | hsa-miR-193a-5p |  | hsa-miR-193a-5p | hsa-miR-193a-5p | ugggucuuugcgggcgagauga |
| 8 | hsa-miR-200b-3p |  | hsa-miR-200b-3p |  | hsa-miR-200b-3p | uaauacugccugguaaugauga |
| 9 | hsa-miR-200c-3p |  | hsa-miR-200c-3p |  | hsa-miR-200c-3p | uaauacugccggguaaugaugga |
| 10 | hsa-miR-296-5p | hsa-miR-296-5p |  | hsa-miR-296-5p | hsa-miR-296-5p | agggcccccccucaauccugu |
| 11 | hsa-miR-30c-1-3p | hsa-miR-30c-1-3p |  |  | hsa-miR-30c-1-3p | cugggagaggguuguuuacucc |
| 12 | hsa-miR-30c-2-3p | hsa-miR-30c-2-3p |  |  | hsa-miR-30c-2-3p | cugggagaaggcuguuuacucu |
| 13 | hsa-miR-3125 | hsa-miR-3125 |  |  | hsa-miR-3125 | uagaggaagcuguggagaga |
| 14 |  | hsa-miR-3142 |  | hsa-miR-3142 | hsa-miR-3142 | aaggccuuucugaaccuucaga |
| 15 |  | hsa-miR-3180-3p |  | hsa-miR-3180-3p | hsa-miR-3180-3p | uggggcggagcuuccggaggcc |
| 16 |  | hsa-miR-3196 |  | hsa-miR-3196 | hsa-miR-3196 | cggggcggcaggggccuc |
| 17 |  | hsa-miR-324-3p |  | hsa-miR-324-3p | hsa-miR-324-3p | cccacugccccaggugcugcugg |
| 18 |  | hsa-miR-331-3p |  | hsa-miR-331-3p | hsa-miR-331-3p | gccccugggccuauccuagaa |
| 19 |  | hsa-miR-338-3p |  | hsa-miR-338-3p | hsa-miR-338-3p | uccagcaucagugauuuuguug |
| 20 |  | hsa-miR-346 |  | hsa-miR-346 | hsa-miR-346 | ugucugcccgcaugccugccucu |
| 21 |  | hsa-miR-370-3p |  | hsa-miR-370-3p | hsa-miR-370-3p | gccugcugggguggaaccuggu |
| 22 |  | hsa-miR-450a-5p | hsa-miR-450a-5p | hsa-miR-450a-5p | hsa-miR-450a-5p | uuuugcgauguguuccuaauau |
| 23 |  | hsa-miR-488-3p |  | hsa-miR-488-3p | hsa-miR-488-3p | uugaaaggcuauuucuugguc |
| 24 | hsa-miR-491-5p | hsa-miR-491-5p |  | hsa-miR-491-5p | hsa-miR-491-5p | aguggggaacccuuccaugagg |
| 25 |  | hsa-miR-516b-5p |  | hsa-miR-516b-5p | hsa-miR-516b-5p | aucuggagguaagaagcacuuu |
| 26 |  | hsa-miR-532-3p |  | hsa-miR-532-3p | hsa-miR-532-3p | ccucccacacccaaggcuugca |
| 27 |  | hsa-miR-574-5p |  | hsa-miR-574-5p | hsa-miR-574-5p | ugagugugugugugugagugugu |
| 28 |  | hsa-miR-602 | hsa-miR-602 |  | hsa-miR-602 | gacacgggcgacagcugcggccc |
| 29 | hsa-miR-625-5p | hsa-miR-625-5p |  | hsa-miR-625-5p | hsa-miR-625-5p | agggggaaaguucuauagucc |
| 30 |  | hsa-miR-628-5p |  | hsa-miR-628-5p | hsa-miR-628-5p | augcugacauauuuacuagagg |
| 31 |  | hsa-miR-629-5p |  | hsa-miR-629-5p | hsa-miR-629-5p | uggguuuacguugggagaacu |
| 32 |  | hsa-miR-647 | hsa-miR-647 |  | hsa-miR-647 | guggcugcacucacuuccuuc |
| 33 |  | hsa-miR-760 |  | hsa-miR-760 | hsa-miR-760 | cggcucugggucugugggga |
| 34 | hsa-miR-874-3p | hsa-miR-874-3p |  | hsa-miR-874-3p | hsa-miR-874-3p | cugcccuggcccgagggaccga |
| 35 |  | hsa-miR-9-5p |  | hsa-miR-9-5p | hsa-miR-9-5p | ucuuugguuaucuagcuguauga |
| 36 |  | hsa-miR-942-5p |  | hsa-miR-942-5p | hsa-miR-942-5p | ucuucucuguuuuggccaugug |
