# Supplementary material for: Deficiency of microRNA-628-5p promotes the progression of gastric cancer by upregulating PIN1
Source: Cell Death Dis. 2020 Jul 23;11(7):559. doi: 10.1038/s41419-020-02766-6 (PMC7378826; doi:10.1038/s41419-020-02766-6)
Supplement: Supplementary file 10 — Supplementary information 10 [file 41419_2020_2766_MOESM10_ESM.doc]

Table S4. Reported microRNAs targeting PIN1

| NM. | miRs | Disease | Reference |
| --- | --- | --- | --- |
| 1 | hsa-miR-140-5p | hepatocellular carcinoma | [18] |
| 2 | hsa-miR-200b-3p | breast cancer | [13] |
| 3 | hsa-miR-200c-3p | breast cancer | [14] |
| 4 | hsa-miR-296-5p | prostate cancer | [15] |
| 5 | hsa-miR-370-3p | esophageal squamous-cell carcinoma | [17] |
| 6 | hsa-miR-874-3p | hepatocellular carcinoma | [16] |
